# Advances in Vision-Based Gait Recognition: From Handcrafted to Deep Learning

**DOI:** 10.3390/s22155682

**Published:** 2022-07-29

**Authors:** Jashila Nair Mogan, Chin Poo Lee, Kian Ming Lim

**Affiliations:** Faculty of Information Science and Technology, Multimedia University, Melaka 75450, Malaysia; 1121116804@student.mmu.edu.my (J.N.M.); kmlim@mmu.edu.my (K.M.L.)

**Keywords:** gait recognition, vision-based, review, deep learning

## Abstract

Identifying people’s identity by using behavioral biometrics has attracted many researchers’ attention in the biometrics industry. Gait is a behavioral trait, whereby an individual is identified based on their walking style. Over the years, gait recognition has been performed by using handcrafted approaches. However, due to several covariates’ effects, the competence of the approach has been compromised. Deep learning is an emerging algorithm in the biometrics field, which has the capability to tackle the covariates and produce highly accurate results. In this paper, a comprehensive overview of the existing deep learning-based gait recognition approach is presented. In addition, a summary of the performance of the approach on different gait datasets is provided.

## 1. Introduction

**Figure 1:**
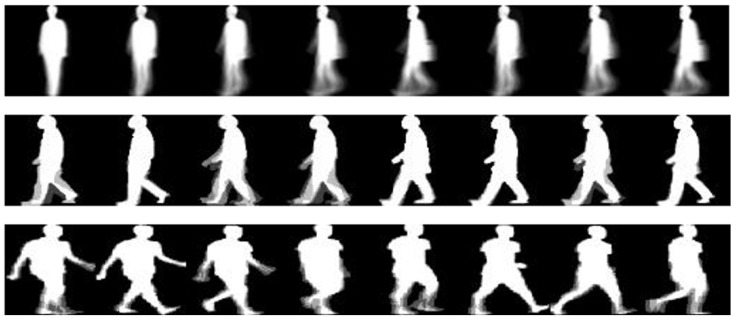
Sample gait images.

Gait is known as an individual’s walking style. Every single gait is different based on the body shape and the distinctive way the body moves. Gait recognition utilizes the aforementioned features to differentiate one person from another. Gait is a passive biometric meaning that it does not require an active response from the people to perform the identification. Other than that, gait is difficult to replicate or disguise as it is a behavior, which takes place casually. Gait is also perceivable at a distance, meaning that a person can be identified from afar [1,2,3,4,5,6]. Some sample gait images are displayed in Figure 1.

**Figure 2:**
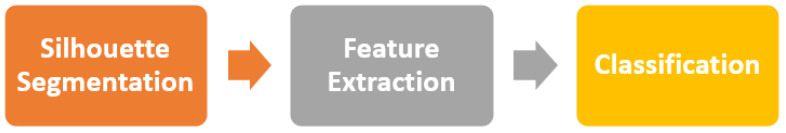
The process flow of the gait recognition system.

The process of gait recognition mainly involves three steps, namely silhouette segmentation, feature extraction, and classification. First, human silhouettes are detected and segmented from the gait sequence. The background subtraction technique is commonly applied to identify the moving human silhouette in a gait sequence. During the feature extraction stage, gait features are extracted from the obtained human silhouettes by using the handcrafted approach. The attained features are then stored as gait signatures. In the classification stage, the subjects are recognized based on the acquired gait signatures. Mostly, k-nearest neighbor (kNN) [7], Hidden Markov Model (HMM) [8] and Support Vector Machine (SVM) [9] are used for gait classification. The flow of the gait recognition process is depicted in Figure 2.

Primarily, handcrafted approaches were used to perform gait recognition. The handcrafted approach can be divided into model-based and model-free approaches. Model-based approaches require a model of the human body to perform the identification, whereas the model-free approach extracts the gait features directly from the gait silhouettes. The model-based approach requires high computational cost compared to the model-free approach. Both the model-based and model-free approaches’ performance were affected by covariates, namely viewing angle, clothing, and carrying conditions.

**Figure 3:**
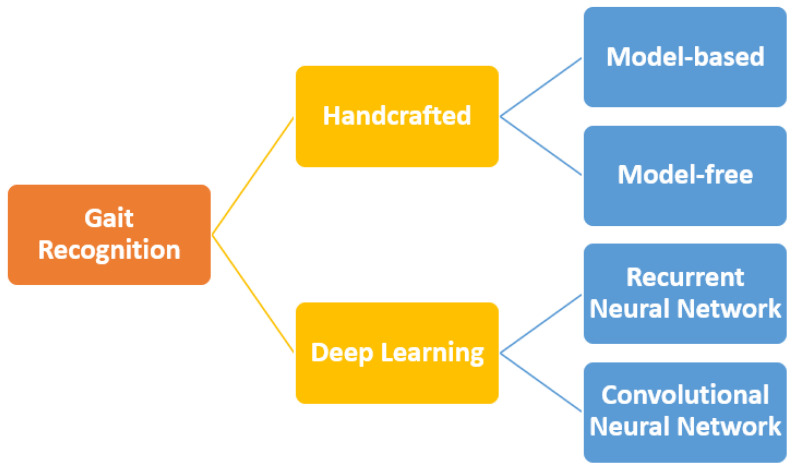
The categorization of gait recognition approach.

Recently, the deep learning approach became popular among the researchers due to the ability to extract features with intricate patterns. The deep learning approach can be used for both feature extraction and classification. The deep learning-based approach has various branches, namely convolutional neural networks (CNNs) [10,11,12,13], recurrent neural networks (RNNs) [14,15,16,17], generative adversarial networks (GANs) [18,19,20,21], and radial basis function networks (RBFNs) [22,23,24,25]. The categorization of the gait recognition approach is shown in Figure 3. This paper discusses the existing handcrafted approach and deep learning approach for gait recognition problems.

## 2. Handcrafted Approach

The handcrafted approach used numerous algorithms to extract manually defined features such as histograms, corners and edges from the gait silhouettes. The handcrafted approach can be categorized as model-based and model free approaches.

### 2.1. Model-Based Approach

The model-based approach models the human body by using stick figures and sets of joints. The movements of the model are tracked based on the length of limbs and trajectories and angles between the joints [26,27,28,29].

In an early paper, Ahmed et al. [30] built a dataset with human skeleton information recorded by using a Kinect camera. Two types of dynamic features were extracted based on the sixteen points (x-axis and y-axis) obtained by the Kinect sensor, namely, the horizontal distance feature (HDF) and vertical distance feature (VDF). HDF was measured by the changes of distances between skeleton joints in the X-axis for each gait cycle. In contradiction, VDF was measured based on the changes in distance between a skeleton joint and the ground in the Y-axis. Four features were proposed under HDF, namely the gap between the left and right shoulders, the gap between the left and right wrists, the gap between the left and right knees, and step length. Step length was determined based on the gap between the right and left ankles instead of the gap between the right and left feet. Mean, skew, and standard deviation were calculated for each of the features for every gait cycle. The mean and standard deviation of six proposed features were calculated for each gait cycle. As for the VDF, six features were proposed, namely the individual’s height, height of right shoulder, height of the right wrist and height of the left and right ankles. The mean and standard deviation of each of the features were computed for every gait cycle. The k-nearest neighbor (kNN) was employed for the classification process.

Wang et al. [31] established a 3D skeleton-based gait dataset, and a walking model was built based on the dataset. The length between joints were selected as a static feature and angles between skeletons were selected as dynamic features. In order to acquire the static feature, two depth thresholds were set as boundaries where the head joint was regarded as the depth of the body. The frames between the boundaries were chosen as reliable frames where the length of the skeleton was calculated at each frame. The components of the static feature vector were then obtained by averaging the length of skeletons. As for the dynamic feature, the angle between a knee to ankle was selected and calculated based on the nearest side to the Kinect. Weighted summation was then applied to merge both the features.

Another 3D skeleton-based gait dataset was built by Sun et al. [32] wherein both the 3D coordinates of skeleton joints, and 2D silhouette images were included. A human walking model was shaped based on the dataset. Static features were acquired from the length of some specific skeletons, while dynamic features were obtained from the angles of swing limbs. In order to extract the gait period, the crossing points were determined and the period between the two points was considered as the gait period. The dynamic time warping (DTW) technique was then employed to measure the distance between the dynamic features from the same individual. Once the final dynamic feature was obtained, the static and dynamic features were combined and fed to the classifier.

Zeng et al. [33] employed deterministic learning theory on lateral-view silhouettes to extract gait features. A 2D five-link biped model is used to extract the side lower-limb joint angles. Gait system dynamics were then identified by using radial basis function (RBF) networks and dynamical RBF identifiers. The acquired information was stored in a constant RBF network, which was used to generate a bank of estimators to depict the trained gait patterns. The obtained gait system dynamics were then inserted in the estimators. The estimators, which contain the gait patterns, were compared, and recognition errors were constructed. The trained gait pattern, which was the same as the test gait patterns, was identified based on the smallest error principle.

Likewise, Deng et al. [34] proposed an algorithm, which extracts spatial–temporal and kinematic gait features by using deterministic learning. A holistic silhouette area and lower-limb silhouette widths of every sequence were extracted as spatial–temporal parameters. A deterministic learning algorithm was then applied on the parameters to compute the spatial–temporal features. Four lower-limb joint angles were extracted from a five-link biped model as kinematic parameters. The deterministic learning algorithm was used to calculate the kinematic features. The classification process was performed by using the smallest error principle.

Sattrupai and Kusakunniran [35] presented a technique by using dense trajectory for gait recognition. In each frame, the gradient magnitude of each pixel in two directions (x, y) were calculated and Eigen-decomposition was applied. A threshold value was then calculated and compared with the gradient magnitude value of the corresponding pixel. The respective pixel was selected as key points if the gradient value is greater than the threshold value. This process was repeated for every frame in the gait video. By using the Lucus–Kanade approach, the flow between two frames was calculated and trajectories connected by 15 frames were generated. Subsequently, trajectory descriptor, histogram of oriented gradient (HOG), histogram of optical flow (HOF), and motion boundary histogram (MBH) were computed and concatenated to describe the key point for every frame. The gait feature was then generated by using Bag of Word (BOW).

In Kovac et al. [36], a frame-based classification was proposed. In order to obtain the gait signals, gait silhouettes were segmented based on body parts and lines of all the body parts were suited through segment midpoints. The lower body parts were divided into thigh bones and shin. A total of 64 gait signals were extracted from the gait video. The frame-based classification was conducted by comparing the gait signals from each video frame. Linear discriminant analysis (LDA) was applied on the features for feature reduction, and kNN was then employed to perform the classification.

Sah and Panday [37] transformed the coordinates acquired by using Kinect into Centre of Body (CoB) coordinates. By doing so, the subject in every frame is rotated to be in the similar direction. Hence, every frame consists of positions of body parts and dimensions of the subject’s body parts. The CoB coordinates were considered as one of the features as the body parts’ positions construct a posture, which is distinctive. Along with that, Euclidean distances of the same joint of subsequent frames were added as the features. The feature vector is constructed based on the aforementioned features. A weighted- kNN was then used to classify the subjects.

Sharif [38] developed a hybrid pre-processing technique to avoid occlusion issues in gait sequences. In the method, hue, saturation, and intensity (HSI) color transformation is first extracted to distinguish between background and the subject. Based on the visual results, a hue channel is chosen, and a weighted logarithmic is executed to enhance the brightness of moving body parts. In order to obtain the enhanced frame, a linear contrast function is implemented on the weighted logarithmic frame. The improved frame is then fed to the Horn–Schunck method to capture the moving regions. Three types of features were then extracted, namely texture, shape, and geometric features. The features were then merged into one vector. Euclidean distance was used for classification.

Table 1 shows the summary of the existing model-based approach. The model-based approach requires hardware sensors (such as Kinect) and complex computations to identify the body parts and joints. Therefore, some researchers opt for the model-free gait recognition.

### 2.2. Model-Free Approach

The model-free approach extracts the gait features directly from the gait silhouettes. Hence, a model-free approach does not require any specific model to carry out the identification process. Due to the simple implementation, the model-free approach is computationally efficient and was popular among the researchers [39,40,41,42,43].

In an early paper, Jeevan et al. [44] presented a new gait representation by using Pal and Pal Entropy to reduce the effect of several covariates, namely carrying condition and clothing. Bounding box and aspect ratio were used to capture a sequence of frames to determine a gait cycle. Gait using Pal and Pal Entropy (GPPE) was then applied on the gait silhouettes to calculate the uncertainty connected with a random variable for every pixel of the silhouette. Principal component analysis (PCA) was used to generate feature matrices for each of the extracted features. The matrices were then fed into SVM for the classification process.

Hosseini and Nordin [45] generated gait representation by using averaged silhouettes theory (Liu & Sarkar, 2004). A gait cycle was determined based on the frames situated among three minimum white pixels in a binary silhouette. The gait representation was then computed by averaging the silhouettes over the number of binary silhouettes of a gait cycle. As for the dimension reduction, Eigenspace transformation was applied on averaged silhouettes. A threshold was used to disregard the trivial eigenvalues. Euclidean distance was employed to measure the distance among the input and trained image set.

Alvarez and Sahonero–Alvarez [46] presented a modified version of GEI representation. By using a random forest algorithm, the head and feet part of GEI were selected as representation. Subsequently, PCA was applied on the modified GEI for feature extraction and dimension reduction. In order to determine the number of components to be fed into the classifier, 2c criteria [47] was used on the extracted features. Linear discriminant analysis (LDA) was then employed to classify the subjects.

In order to preserve the temporal information over the gait cycle, Luo et al. [48] developed an approach that accumulates the frame difference energy image. At first, GEI was computed by averaging the frames over a gait cycle. Then the frame difference energy image was computed by combining forward and backward frame difference energy images of the first six frames in a gait cycle. The accumulated frame difference energy image (AFDEI) was then attained by using a weighted average method. In order to preserve the static feature and temporal feature, GEI and AFDEI were combined as the final feature. Nearest neighbor was employed as the classifier.

Arora and Srivastava [49] presented a period-based approach named Gait Gaussian Image (GGI) for gait recognition. Region of interest and bounding box were used to extract the gait silhouettes. GGI was calculated for every pixel of an image over frames in a gait cycle. The Gaussian function was applied on the obtained vector to perform the fuzzification process. The acquired value of a pixel was then multiplied by its respective pixel and averaged over all the values, which generated a composite image. Subsequently, nearest neighbor and Euclidean distance were employed to classify the subjects.

Fathima et al. [50] proposed an algorithm that extracts six different angles from head to toe along with the height and width of the subject silhouette. The width and height were computed based on the bounding box and the centroid point of each human body whereas the angles were measured based on the deviation of reference partitioned planes. Support vector Machine (SVM), kNN, and relevance vector machine (RVM) were used to classify the subjects, and the performance was compared in terms of angle variation and cloth variation.

Later, Rida et al. [51] incorporated statistical dependency (SD) and globality–locality preserving projections (GLPP) to eliminate the effects of intraclass variations. At first, SD was applied on gait energy image (GEI) to calculate the scores of usefulness of every feature. Subsequently, the features were ranked based on the scores given. GLPP was then applied on the feature for dimension reduction. GLPP is employed to also conserve the geometric structures and to extract the interclass and intraclass differences. The classification was performed by using 1-NN.

Wang et al. [52] presented a method, which utilizes Gabor wavelets for gait recognition. GEI was generated by averaging the frames over a gait cycle. In order to generate Gabor feature vectors, a Gabor wavelet was applied in five different scales and eight orientations on the attained GEI. Then, the dimension reduction of the feature vector was performed by using a 2D PCA (2D2PCA). The 2D2PCA was employed due its ability to reduce the distance within class and increase the distance among classes. SVM was adapted to conduct the classification process.

A supervised feature selection method for gait recognition was developed in Rida et al. [53]. Each row of GEI was used to estimate the horizontal motion and was considered as a feature unit. Then the sum of each rows’ intensity and all rows’ intensity was used to calculate the dimensional intensity. A mask that was able to pick the important features from GEI was identified for three types of covariates, such as normal walking, clothing and carrying condition. By using the “AND” operation, the masks of the same covariate were combined. Subsequently, all three types of masks were combined by using the “AND” operation and multiplied with GEI to produce the masked-GEI. PCA and multiple discriminant analysis (MDA) were applied on the masked GEI to generate the final gait feature.

Similarly, Rida [54] proposed a method that selects the dynamic human body parts automatically from GEI images. Horizontal motion was first estimated by applying Shannon entropy in each row of the GEI and generated motion-based vectors. Then body parts were segmented by using group-fused lasso by identifying the shared change-point across all the obtained motion vectors. Canonical discriminant analysis (CDA) was employed for feature dimension reduction and to avoid overfitting issues. The classification was performed by using NN.

Mogan et al. [55] integrated motion history image (MHI), binarized statistical image features (BSIF) and histograms of oriented gradients (HOG) to extract the temporal patterns and direction of gait sequences. In order to conserve the changes in motion at every pixel, MHI was applied on the input image. BSIF was employed where the MHI images were convolved with a set of pre-learned filters and binarized. The binarized features were then concatenated. Then, the BSIF images were divided into several cells. The gradient of every pixel in the cell was calculated. The pixels in the cell cast a vote for a histogram channel based on the obtained gradient value where the channel was evenly spread from 0° to 180°. Block normalizations were then applied to generate the gait descriptor. The classification was performed by using Euclidean distance and majority voting.

Later, Mogan et al. [56] proposed a method named histograms of temporal gradients (HTG) for gait recognition problems. A set of filters that was learned by using natural images were used as a convolution kernel. A set of feature maps were generated by convolving input images with the set of pre-learned filters. The obtained feature map was then segmented into several regions and gradient of each pixel in the region was calculated. Based on the gradient, the bin number to which the pixel belongs was determined. The pixel of the current and subsequent frames cast a vote in the matrix of gradient patterns. The matrix of each region was vectorized into histograms. The histograms of all the regions were then reorganized into a regional histogram. Time normalization and block normalization were applied on the regional histograms. All the histograms of a gait cycle were concatenated into a final histogram. Majority voting with Euclidean distance was employed during the classification stage.

Table 2 shows the summary of the existing model-free approach. Although the model-free approach does not require the creation of the body model, it still requires manual feature engineering, which is susceptible to performance degradation when there is an appearance change due to covariates such as viewing angles, clothing and carrying condition.

## 3. Deep Learning Approach

Deep learning approaches have the ability to learn commendable features from the inputs without having them specified. Hence, the current research is mostly based on deep learning approaches. The deep learning approach extracts high-level gait features from raw input data by learning over a large amount of data. The deep learning approach can be categorized into RNNs and CNNs.

### 3.1. Recurrent Neural Networks

RNN has recurrent connections whereby it is able to obtain the temporal information in sequences. The connections with prior stages enable RNN to memorize the past information and capture the circumstantial dependency of the consecutive data. RNN has a built-in memory block to store the relations between the past and current stages.

McLaughlin et al. [57] incorporated CNN, RNN, and temporal pooling layer in a Siamese network for video-based human re-identification. The color and optical flow gait sequences were fed into the network. In CNN, max-pooling and hyperbolic-tangent activation functions were applied on each layer to produce feature maps. In the last layer of CNN, the feature maps were vectorised into feature vectors. The acquired feature vector was fed into RNN and the Tanh function was used in time-steps. In the temporal pooling layer, the long-term motion information is acquired by accumulating the information over all the time steps. The dropout technique was employed in both the CNN and recurrent layer to reduce overfitting. Euclidean distance was applied to classify the subjects.

Likewise, Varior et al. [58] presented a Siamese long short-term memory (LSTM) network wherein the competency of local features is improved by utilizing the contextual information for person re-identification. The Siamese network consists of a pair of LSTM networks. The networks take input data in a parallel way and learn an embedding process where the similar image pairs were kept close while unrelated ones were kept away. The inputs were alienated into various horizontal rows as a spatial sequence in order to produce view-invariant gait representations. Local maximal occurrence and color name features were then extracted from the input images. The obtained features were fed into the single-layer LSTM network to acquire the hidden representations and the loss was computed by using contrastive loss function. Root mean square propagation (RMSProp) was employed to update the weight parameters.

Li et al. [59] incorporated skeleton data and LSTM network to generate discriminative features of sequences with varying length. The network comprises two recurrent hidden layers, one fully connected layer and a softmax layer. A sequence of skeleton data was fed into the network at one timestamp in chronological order. The output of the first layer at each timestamp was fed to the second layer. In order to avoid overfitting, the dropout technique was applied. The network parameters were updated by using sigmoid function. The classification was performed by using a softmax function.

Zhang et al. [60] integrated CNN with the LSTM attention model to extract spatial–temporal information. Along with that, a gait-related loss function, termed angle center loss, was presented. The inputs were segregated into four rows and fed into separate CNNs. The CNN comprises three convolution layers, three pooling layers, two batch normalization layers, and one fully connected layer. A dropout technique was used to reduce overfitting. The local features were then extracted in every CNN and were concatenated to produce the final descriptor. Angle center loss with SGD were applied in the CNN. The LSTM attention model was then used to generate the temporal attention scores for frame-level features. The attained scores were then averaged by using attention weight for every part.

A time-based graph long short-term memory (TGLSTM) network was presented by Battistone and Petrosino [61]. The network utilized structured data and temporal information for gait recognition. The network comprises fully connected layers and LSTM in alternate order. At first, the graph for each frame was built by skeletonisation and polygonal approximation techniques. The graph extracted the changes of shape and size that occurred over time. The obtained skeleton graph was then fed into the TGLSTM model and produced the action denoted by the graph. The last fully connected layer mapped the output classes and fed them to the softmax layer for classification. ReLU activation function was applied in the fully connected layers. Gaussian noise was added to the LSTM weights to avoid overfitting issues.

Tong et al. [62] incorporated CNNs and LSTM (CNNs–LSTM) to extract the spatial and temporal information. The CNNs consist of three convolutional networks, comprised of convolution layer, activation function, and pooling layer. Gait contours were acquired by using a heuristic method and fed to the triple CNN network as inputs. CNN was employed to capture the spatial features. The obtained spatial feature maps were then fed to the LSTM layer, where the temporal features were extracted. Euclidean distance was employed to calculate the distance between two gait sequences. The output of the LSTM layer was sent to lose the layer, where a triplet loss function was adopted to train the network.

Likewise, Wang and Yan [63] developed an LSTM model with added convolutional layers, termed Conv-LSTM. A frame-by-frame GEI (ff-GEI) gait representation was presented along with the model. The network comprises three convolutional and pooling layers, a fully connected layer, three LSTM layers and a softmax layer. The ff-GEIs were fed into the three sets of convolutional and pooling layers. The obtained feature maps from convolutional layers were reconstructed into a graph through the fully connected layer. The output of the fully connected layer was transformed into vectors and fed to the LSTM layers to be further improved. Max-pooling was employed in the pooling layers. The ReLU function was used in the convolutional and fully connected layers. A dropout technique was employed in the fully connected layer and LSTM layer. Thereafter, the softmax function and cross-entropy were applied in the classification process.

Liu et al. [64] proposed skeleton gait energy image (SkeGEI) as a new feature to be extracted along with relative distance and angle (DA) for gait recognition. A CNN–LSTM network was developed in this work. The CNN network was composed of three convolution layers and three max-pooling layers. The LSTM network comprises two LSTM layers. Human skeleton coordinates obtained from the Kinect camera were converted into a skeletal grayscale image to produce the SkeGEI. As for the DA features, joint angle and joint distance were measured. The distance between five joints of hands and legs were obtained as distance features. The angles of two arm joints and three leg joints were considered as angle features. The SkeGEI features were sent to CNN network to extract the spatial feature, whereas DA features were sent to LSTM to capture the temporal feature. Both the features were then concatenated in a fully connected layer through backward propagation. The SVM classifier was employed for classification stage.

Later, Zhang et al. [16] incorporated Autoencoder and LSTM to extract dynamic and static gait features. Along with that, a frontal view gait (FVG) dataset under several covariates were collected. In this work, three types of features were extracted, such as the pose feature, canonical feature, and appearance feature. The pose feature defines the location of the body parts. As for the canonical feature, the special features of the subject’s body were described. A subject’s clothing was described in the appearance feature. An encoder–decoder network was employed to detach the aforementioned features for every frame. Three different loss functions were applied in the network, namely cross-reconstruction loss, pose similarity loss, and canonical similarity loss. Pose features over time were captured as dynamic features, and canonical features were averaged as static features. The dynamic feature was captured by using a multi-layer LSTM, and the output was averaged to generate the final gait feature. Incremental identity loss function was used to learn the LSTM network.

The RNN with bidirectional gated recurrent units (Bi-GRU) was adopted to capture the temporal information from human pose sequences in Hasan and Mustafa [65]. The network consists of two Bi-GRU layers and a softmax layer. Their work included several feature extraction processes, namely raw 2D body joints, joints angular trajectory, temporal displacement and body-part length. The aforementioned processes extracted four types of features in order to obtain more spatial–temporal information. In the feature level fusion, several features of the same frame were concatenated before being fed into the network. The Adam optimization technique [66] was applied to optimize the network. The loss was computed by using fusion of center loss and the softmax loss function.

Li et al. [67] incorporated a pre-trained human mesh recovery (HMR) network and a skinned multi-person linear (SMPL) model for end-to-end gait recognition. Shape and pose features were extracted from the RGB gait sequence by fitting the SMPL model. The parameters of the SMPL model were then extracted, and a 3D human body mesh was estimated by the pre-trained HMR. The HMR was then fine-tuned by using reconstruction loss between rendered silhouettes and silhouette masks. By doing so, the HMR network was able to adapt to different numbers of subjects in datasets through transfer learning. The obtained features were then sent to recognition networks separately. The shape feature was averaged by using the averaged shape feature. As for the pose feature, LSTM and CNN networks were employed to extract the spatio–temporal features. By using a three-layer LSTM, an average of the LSTM output sequence was considered as a pose feature. Subsequently, the CNN with three convolution layers and a fully connected layer was used to extract the pose features as well. Triplet loss was used during the training stage and Adam was employed as the optimizer.

Wen and Wang [68] developed a residual long short-term memory network for cross-view gait recognition (CVGR-RLSTM). The network was made up of three modules, namely a residual block and two LSTM blocks. The RLSTM was fed with ff-GEIs to capture the time-wise gait information. The spatial information of the inputs was enhanced by the residual block. The spatio–temporal information was then extracted by the LSTM blocks respectively. Two distinct layers were added to the network, namely convolutional layers and batch normalization layers. The purpose of the convolutional layer was to connect the independent channels in the input. The batch normalization layer was added to avoid vanishing gradient problems.

Table 3 shows the summary of the existing RNN approach. RNN consists of memory and gating mechanism that store the former inputs in order to learn the relation with the current input. The network uses a backpropagation process to update the weights. Because RNN is based on timestamp, the process of backpropagation through time requires huge computational time and power.

### 3.2. Convolutional Neural Networks

CNN is a popular network among the researchers due to its high discriminative power compared to other networks. Other than that, CNN-based models offer an efficient way to extract features from both images and frame sequences. Typically, CNN consists of convolution layers, pooling layers, normalization layers, and fully connected layers.

Song et al. [69] incorporated two CNNs into one network to combine the gait- segmentation process and gait-classification process. A multichannel fully convolutional network (FCN) [70] was developed in the gait-segmentation network to segment the gait sequences. FCN was composed of seven convolution layers to extract the gait features and one deconvolution layer for the final segmentation process. RGB gait sequences were concatenated and fed into the FCN to perform segmentation. The obtained gait features were integrated into gait templates to be sent to the recognition network. The gait recognition network contains the multi-scale context-aware network (MSCAN). The model comprises four convolution layers and two fully connected layers. The softmax function was employed to classify the subjects and the network was trained by using SGD.

Zhu et al. [71] presented a CNN-based network called LFN, which includes the pre-processing of the input image. The network was composed of three convolution layers and one fully connected layer. The binarization silhouette was fed into the network and processed in the convolution layer, and the activation and pooling layer. Then the data was copied into three images to be fed into three convolution layers. The three convolution layers extract the minute details of the input image. All the extracted features were then combined and fed to a fully connected layer. The ReLU activation function was used in the convolution layers. The max operation was applied in the pooling layers. In order to prevent overfitting, the dropout technique was applied in the fully connected layer. Negative log likelihood loss (NLLLoss) was employed as the loss function.

Later, Su et al. [72] proposed center-ranked loss function where the positive input was pulled closer to a specified threshold, and the negative input was pulled away from the threshold. A network with six convolution layers, three max-pooling layers and one fully connected layer was presented. The features were extracted, and the dimension was reduced through the convolution layers and max-pooling layers. The max-pooling was applied on the feature maps obtained in the last convolution layer, in order to integrate the spatial and temporal features. The fully connected layers were used to combine all the features into a gait representation. The proposed center-ranked loss was applied to train the network.

A traditional CNN added with a feature extraction layer by using Gabor filters was presented in Wen (2020) [73] for gait recognition. The network consists of three convolution and pooling layers. The Gabor filter was injected at the input layer to pre-process the gait images and extract the gait features. Convolution and pooling layers were employed to further extract the gait features. The max-pooling technique was used in the pooling layers. Unlike the traditional CNN, a KNN classifier was used in this work to classify the subjects. The metric learning-based algorithm was applied in the classifier, where the initialisation of the number of subjects was not required. Other than that, the Mahalanobis distance was used to keep the gait features of different subjects away from each other.

Fan et al. [74] constructed a part-based network, which boosts the performance of gait recognition. The network comprises two components, namely the frame-level part feature extractor (FPFE) and the micromotion capture module (MCM). The FPFE consists of three blocks, which are composed of two focal convolution layers (FConv) and one pooling layer each except the third block. The FPFE was fed with a sequence of gait silhouettes. Spatial features were extracted through the FPFE component. The output of the FPPE was then fed into horizontal pooling (HP) to divide the feature maps into pre-defined horizontal partitions. By doing so, different gait patterns were extracted from each of the partitions. The part representation matrix (PR-Matrix) was then obtained by transforming the part-level feature vectors. Each of the PR-Matrix represents the gait changes of each of the partitions. MCM was then employed to combine all the PR-Matrix to produce spatio–temporal features for the recognition process. Triplet loss was used to train the network and Adam optimizer was employed in the model training.

Subsequently, Hou et al. [75] presented a gait lateral network (GLN) to extract compact and discriminative information from gait silhouettes. The set-pooling and max-pooling order in the network [76] was modified and used as the backbone in this work. The network’s layers were divided into three stages. The first stage consists of two convolution layers where the gait silhouettes were transformed into internal features. Both the second and third stages were composed of two branches, which extract the set-level and silhouette-level features. The silhouette-level and set-level features were extracted by using max-pooling function. The extracted features were then combined in a top-down manner by using the pooling function. A compact block was introduced to reduce the dimension of the gait representations without affecting the accuracy. The compact block was composed of one-batch normalization, ReLU activation function, dropout technique, fully connected layer, and another batch normalization. The network applied two types of training strategies, namely lateral pretraining and global training. The lateral pretraining employed triplet loss whereas the global training used the sum of triplet loss and cross-entropy loss. The lateral pretraining was conducted to attain a sensible initialization for lateral connections. On the other hand, global training was carried out to train the whole network, and SGD was employed as the optimizer.

A sequential convolutional network (SCN) was proposed in Ding et al. [77] to capture the behavioral features from a gait sequence. A sequence of gait silhouettes was fed into the network. The SCN was composed of three groups of transition and gait blocks where the features of each frame were extracted. The gait block consists of the behavioral information extractor and the convolutional block. The convolutional block comprises convolution layers, max-pooling layers, and Leaky ReLU function. The behavioral information extractor was used to attain the motion information of a gait sequence. Then the obtained information was sent to the convolutional block to capture the temporal information. A multi-frame aggregator was then employed to combine and transform all the obtained features of a sequence into a sequence-level feature. Batch All (BA+) triplet loss was applied to train the network, and Adam was employed as the optimizer.

Yoo and Park [78] developed a skeleton silhouette-based disentangled network for view-invariant gait recognition. Three input images were sent to the network. Two inputs are from the same subject with different views and conditions, and the other was from a different subject. A total of 18 body joints were extracted from the skeleton by using OpenPose [79] and refined by using Posefix [80]. Then the final silhouette image was generated. The features were disentangled into pose features in order to learn dynamic features. Eight images were randomly chosen from a gait sequence to be encoded. The output of the encoder was concatenated to generate the static and dynamic features. A total of four losses were employed in this work, namely reconstruction loss, gait consistency loss, canonical consistency loss, and identification loss. The reconstruction loss was used to make sure the canonical information stays the same across the frames in a video. The gait consistency loss was applied to enforce the consistency of pose features and dynamic features. The canonical consistency loss was employed to have the constant features across the video frames. The identification loss was used to preserve the individuality of the feature, and Adam was applied as the optimizer.

In Jia et al. [81], CNN was incorporated with an attention mechanism to concentrate on the discriminative area to perform gait recognition. The CNN was used to transform the RGB inputs into feature vectors as the input of the attention model required to be vectors. The CNN consists of three convolution layers, two max-pooling layers, and one fully connected layer. The convolution layers were used to extract the features, and the pooling layers were employed to reduce the dimension of the features. In the fully connected layer, the output of the second pooling layer was transformed into a vector. The output was then fed into the attention model in temporal order for the classification process. The attention model comprises two similar encoder layers and one decoder layer to encode and decode the information.The linear operation was applied to transform the output, and the softmax function was used to classify the subjects.

In earlier days, most of the works used GEI as the input due to its high accuracy. Shiraga et al. [82] developed a network consisting of two sequential triplets of convolution, pooling and normalization layers, two fully connected layers, and a softmax layer. The frames were averaged over a gait cycle to obtain GEI and were fed into the network. The ReLU activation function was used in all the convolution layers and the first fully connected layer with dropout. In the pooling layer, max-pooling was used, and in the normalization layer LRN was applied. The learning of the features was performed by minimizing the cross-entropy loss. The SGD algorithm was used to update the set of weighting parameters, and the softmax function was applied to classify the subjects.

Yeoh et al. [83] presented a CNN method for clothing-invariant gait recognition, which extracts the discriminative changes of gait features from GEI input images. The network comprises three convolutional layers, three max-pooling layers, two fully connected layers, and a softmax layer. The convolution layers generated a number of feature maps separately. The ReLU activation function was applied in all the convolution layers. LRN was employed after all the convolution layers. As for the overfitting issue, dropout was used after the fully connected layers. The model was trained by using SGD, and the weights were initialized by using Gaussian distribution.

Wu et al. [84] presented a deep CNN, which measures the similarities between a pair of inputs. Two different architectures were proposed, namely local@bottom (LB) and mid-level top (MT). The main difference between the aforementioned networks was seen when the similarities of a pair were calculated. Pairs of GEIs were the inputs of all three networks. The LB consists of three convolution layers, two normalization layers and two spatial pooling layers, whereas the MT was composed of two LB networks. In the LB network, the differences were calculated at the bottom layer, and the patterns of the acquired differences were learned through the remaining convolution layers. Contrarily, the MT network learns the features before calculating the differences of the GEI pair. The ReLU function was applied on the convolution layers. The classification was performed by the softmax function, and the networks were trained with logistic regression loss.

In Takemura et al. [85], two CNN architectures with triplet ranking loss for cross-view gait recognition was developed. The first architecture, 3in was made up of three parallel CNNs while, 2diff consists of two parallel CNNS. The 3in network was used for larger view difference whereas the 2diff network was used for smaller or no view difference. Both the networks consist of three convolution layers, two normalization layers, two pooling layers, and a fully connected layer. Triplet GEIs, such as positive, negative, and query, were the inputs of 3in network. Two images acquired by subtracting the positive or negative from the query were the inputs of 2diff network. In all the convolution layers and the fully connected layer, the ReLU activation function was employed. The max-pooling technique was used in the pooling layers. Local response normalization (LRN) was employed in the normalization layer. The dropout technique was used in the fully connected layer. The weight and bias parameters were updated by using SGD algorithm.

Tong et al. [86] developed a triplet-based CNN with embedded learning. The CNN network comprises three convolution layers, three normalization layers, three pooling layers, and one fully connected layer. Three GEI inputs were fed into the network, namely positive, query, and negative. The positive and query inputs were from the same subject but taken under different viewing angles, and the negative input was selected from a different subject but under the same viewing angle as query input. The gait features were extracted from each of the inputs separately, and their identity was predicted by using classification loss function. The Euclidean distance was employed to calculate the distance between the three inputs. The triplet loss function was employed to train the network.

Subsequently, Alotaibi and Mahmood [87] proposed a deep CNN that is less sensitive to occlusions and variations. GEI was used as the input of the network. Their network contains four convolution layers and four pooling layers with eight feature maps each. In each convolution and pooling layer, eight convolutional filters and eight sub-sampling maps were randomly set. The backpropagation learning algorithm was applied to train the layers, and SGD was used to minimize the cost function.

Wu et al. [88] presented a pixel-level feedback weight CNN that emphasized on the significance of different body parts. The CNN comprises two 2D convolutional and max-pooling layers and a fully connected layer. GEI, as the input of the network, was fed into the convolutional layer. In CNN, the features were extracted by computing the pixels on based on the region. The attained region-based features were merged and fed into the fully connected layer to produce the final feature vector. The Euclidean distance was applied to classify the subjects.

A joint intensity transformer network was proposed in Khan et al. [89] for gait recognition under clothing and carrying conditions. The method comprises a joint intensity metric estimation net (JIMEN), joint intensity transformer (transformation module), and a discrimination network (DN). The GEI image was selected as the input of the network. The JIMEN network consists of four convolutional layers, three max-pooling layers, and four deconvolutional layers. The DN contains three convolutional and max-pooling layers and a fully connected layer. The ReLU activation function was applied on all the convolutional layers and the fully connected layer. The JIMEN was employed to estimate the joint intensity metric, and the DN was used to learn the spatial metric. The SGD algorithm was used to update the weights and biases, and the SVM classifier was used to classify the subjects.

Wu et al. [90] constructed a feedback weighted capsule network to identify if a pair of images were from the same subject despite the viewing angles and carrying conditions. The capsule model was separated into three parts, namely to update the input image with a pixel-level feedback weights matrix, extract of gait features with a convolutional network, and produce the similarity of the image with an improved capsule. GEI was fed into the network to pre-train the model several times. The network parameters, gait features, and feature vector extracted from the gait feature were then obtained. The weight matrix of each of the receptive fields were trained based on the feature vector, and the input layer was updated by the weighted receptive field. The convolutional network consists of three convolution layers, pooling layers, and batch normalization layers. The ReLU activation function was employed in all the convolution layers. Max-pooling was used in the pooling layers. In the convolutional network, gait features were extracted. The improved capsule transformed the gait features into vector neurons and combined and predicted the similarity. In order to avoid overfitting, the dropout technique and batch normalization were applied.

Xu et al. [91] presented a unified CNN architecture comprising a pairwise spatial transformer network (PST) and recognition network (RN). The PST consists of a localization network, grid generator, and sampler. The localization network was composed of two convolution layers, two pooling layers, and two fully connected layers. Max-pooling was applied in the pooling layers and the ReLU function was employed in the convolution layers and the first fully connected layer. Local response normalization was used before the pooling layers. The pair of GEIs (probe and gallery) were fed into the localization network. The last fully connected layer in PST regressed the transformation parameter vector. The vectors were then fed into the grid generator, where a warping field was produced. Both the probe and gallery GEIs are transformed into their intermediate view. The sampler transformed the input GEI pairs. The RN contains three convolution layers, two max-pooling layers, and a fully connected layer. The ReLU function was applied in the convolution layers and fully connected layers. LRN was applied before the pooling layer.The dropout technique was used in the fully connected layer. The transformed GEI pairs were then fed into RN to identify the dissimilarity of the pair by using the L2 norm.

In Wang and Yan [92], a non-local neural network (NLNN) was employed to perform the gait recognition. GEIs and pairs of GEI were used as inputs of the network. Pairs of GEI, which were positive and negative, were sampled from the GEIs. The NLNN consists of three CNN modules, which comprises a convolution layer, pooling layer, and normalization layer. The pooling layer and normalization layer of the third module was removed and added dropout to avoid overfitting. The ReLU function was applied in the convolution layers. In each of the CNN modules, non-local features were extracted from the GEI pairs. The third layer split the feature map into three clusters, namely a microdynamic region, a weakly dynamic region and a strongly dynamic region. The regionalized features were then fed to three softmax classifiers to classify the subjects.

Balamurugan et al. [93] presented a deep CNN for gait recognition. The CNN network comprises four convolution layers, four max-pooling layers, a fully connected layer and a softmax layer. GEI was used as the input of the network. The features were extracted in the convolution layers and feature maps were produced. The ReLU function was then applied on the feature maps. A softmax classifier was employed to classify the subjects. The backpropagation learning algorithm was used to train the network and stochastic gradient descent with momentum (SGDM) was used as the optimizer.

Elharrouss et al. [94] proposed a network consisting of two CNN models. The first CNN model was developed to estimate the image captured angle, and the second model was constructed to recognize the gait. The angle estimation model consists of two convolution layers, two max-pooling layers, one flattened layer, and one fully connected layer. The gait recognition model consists of three convolution layers, three max-pooling layers, one flattened layer, and two fully connected layers. GEI was generated from a gait sequence fed into the angle estimation model. Based on the input image, the angle was determined. The output of the angle estimation model was then fed into the gait-verification model to identify the gait. The parametric rectified linear unit (PReLU) activation function was employed in both the models. The dropout technique was used to avoid overfitting issues. Both the models were trained by using cross-entropy.

Similarly, Xu [95] developed a deep large-margin nearest neighbor (DLMNN) method for gait recognition. CNN was employed as the network backbone. The CNN was composed of five convolution layers, five pooling layers, and one fully connected layer. The ReLU function was applied on all the convolution layers except the last convolution layer. Max-pooling was used in the first four pooling layers, and the average pooling in the last pooling layer. Three GEI inputs were used in the network, namely positive, objective, and negative. The positive and objective inputs were from the same subject, and the objective and negative inputs were from different subjects. These triplet inputs were fed into the CNN and mapped into feature space. The DLMNN loss function applied on the features to bring the features of the same subjects together and pull the different subjects away. Nearest neighbor was applied to classify the subjects. SGD was used to train the network with DLMNN loss function.

Mogan et al. [96] utilized the transfer learning technique to incorporate the pre-trained DenseNet-201 model and multilayer perceptron. The pre-trained DenseNet-201 model comprises a convolution layer, a max-pooling layer, four dense blocks, and three transition layers. The multilayer perceptron consists of two fully connected layers, two batch normalization layers, and a classification layer. The fine-tuning technique was applied on the pre-trained DenseNet-201 model to extract the gait features of GEI. The multilayer perceptron was then used to identify the relationship between the learned gait features and class labels. and the softmax function, and categorical cross-entropy function were employed to classify the subjects. The leaky-ReLU activation function and dropout technique were used in both the fully connected layers. Moreover, the early stopping technique was utilized to prevent the network from overtraining.

Some of the previous works applied different techniques to produce new gait representations, which played an important role in improving the accuracy. Wang et al. [97] designed a new gait representation by using three consecutive gait silhouettes, termed trituple gait silhouettes (TTGS). Furthermore, a multichannel CNN network, which accepts a set of sequential images in parallel, was developed. The network comprises five convolution and pooling layers, one fusion layer, one fully connected layer, and an output layer. The TTGS were fed into the network in a parallel way in order to preserve more dynamic information. In the pooling layer, max-pooling was employed. The ReLU activation function was applied in all the convolution layers and fully connected layers. The dropout technique was used in the fully connected layer to avoid the overfitting issue. The softmax function and cross-entropy were applied to classify the gait features. The SGD algorithm was used to update the weights and biases.

Wang and Zhang [98] presented two types of two-branch CNN (TCNN) networks, namely middle-fusion TCNN and last-fusion TCNN along with new gait representation termed multi-frequency GEI (MF-GEI). Both the TCNNs consist of five convolution and pooling layers, a concatenation layer, and a fully connected layer. GEIs and the corresponding MF-GEIs were fed into two branches and different convolution filters were applied. The difference between the two TCNNs was the placement of the concatenation layer where the concatenation of feature maps took place. The weight and bias parameters were updated by using the SGD algorithm. SVM classifiers were employed for the classification process. In order to prevent overfitting issues, early stopping and dropout techniques were used in this work.

Later, Chao et al. [76] exploited gait as a deep set to produce a method that was resistant to frame transformations. A set of gait silhouettes were fed into several convolution layers to extract frame-level features from each silhouette. Based on set pooling (SP), a set of obtained frame-level features were projected into a set-level feature by using a permutation function. Horizontal pyramid mapping (HMP) was proposed to learn the discriminative representations from the set-level feature. A multilayer global pipeline (MGP) was proposed to obtain different levels of set information and to produce the final feature maps. The network was trained by using the Adam optimizer. A combination of cross-entropy loss and triplet loss were employed in this work.

Liu and Liu [99] incorporated a densely connected convolutional network (DenseNet) and a stacked convolutional autoencoder (SCAE) called two-stream neural network (TS-Net). The network consists of two-stream networks, namely a mainstream network and an auxiliary stream network. The mainstream network was employed to extract the dynamic features whereas the auxiliary stream network was used to extract the static features. The mainstream network was based on DenseNet, which comprises one input layer, four dense blocks, and three compression layers in between the dense blocks and one output layer. The input layer consists of one convolution layer and one max-pooling layer where the dynamic features were extracted and the input image was resized. Each of the dense blocks contains two convolution layers where batch normalization and the ReLU function were applied. The output image’s size of each dense block was reduced to half by using convolution and average pooling kernel. The extracted dynamic and static features were integrated, and the final gait feature was attained by applying the average pooling layer and the fully connected layer in the output layer. The dropout technique was applied in the fully connected layer to reduce overfitting. The Sigmoid function was employed to measure the similarity between the gait images. Binary cross-entropy loss function was used to calculate the loss, and the Adam optimization was used to update the parameters.

Chai et al. [100] suggested a framework that was able to be incorporated with the existing networks. There were several networks that were integrated with the proposed framework. The features were extracted by using the backbone network and sent to two different branches. HPP was applied on the first branch, and the pooling operation was used on the second branch. The pooling operation was performed in order to obtain the view classification information. The projection matrix was chosen based on the predicted view. For every feature calculated in HPP, the corresponding matrix was multiplied to obtain the final view-invariant feature. Cross-entropy loss and triplet loss were applied in this network.

Wang and Yan [101] presented an architecture based on CNN ensemble (GCF-CNN), which includes primary classifiers and secondary classifiers for gait recognition. Random sampling with replacement strategy was used to construct GEIs in a series of different training sets. The primary classifiers contain several convolutions, ReLU, and pooling layers with one fully connected layer. Similar to the primary classifier, the secondary classifier consists of several convolutions, ReLU, and pooling layers, one fully connected layer, and a softmax layer. Max-pooling was applied in all the pooling layers. The series of training sets was fed into the primary classifiers. During the primary classifiers, different gait features were extracted. The output of the fully connected layer was fed into the secondary classifier where all the gait features were combined. SGD was used to update the weights and biases.

Table 4 shows the summary of the existing CNN approach. Various CNN architectures were proposed to encode the spatial features for the gait representations. Among the existing works, GEIs had been widely used as the input for the CNN.

## 4. Gait Datasets

There are several publicly available gait datasets. Among the gait datasets, the CASIA-B dataset [102], the OU-ISIR Treadmill Gait dataset D [103], the OU-ISIR Large Population dataset [104] and the OU-ISIR Multi-View Large Population dataset [105] are widely used in the deep learning approach due to the various viewing angles and different walking conditions.

### 4.1. CASIA-B

The CASIA-B dataset is made up of 124 individuals including 93 males and 31 females. The dataset is known as the large multi-view gait database, as the gait sequences were recorded from eleven views at a difference of 18°. Each gait sequence consists of two to three gait cycles. The viewing angle, clothing, and carrying condition are the variations considered while recording the gait sequences. The gait sequence for each individual was captured six times under normal walking, twice for both the clothing and carrying condition.

### 4.2. OU-ISIR Treadmill Gait Dataset D

The OU-ISIR Treadmill Gait dataset D (OU-ISIR D) consists of 185 individuals with 370 gait sequences recorded from the lateral view. The dataset was built to evaluate how the gait varies over a number of periods in a gait sequence. The dataset was divided into DBhigh (steady walking style) and DBlow (fluctuated walking style). Both the subsets contain 100 individuals each in which 15 of them overlapped. A normalized autocorrelation (NAC) of size-normalized silhouettes for a temporal axis was used to quantify the gait fluctuation.

### 4.3. OU-ISIR Large Population Dataset

The OU-ISIR Large Population dataset (OU-LP) contains 4016 individuals in which 2135 of them are males and 1872 are females. The individuals were aged 1 to 94 years old. The dataset consists of two subsets, namely sequence A and sequence B. In sequence A, every subject has two sequences (gallery and probe), and in sequence B one sequence each. The subsets were further divided into five sets based on the captured angle such as 55°, 65°, 75°, and 85°, and all angles. Sequence A was created for assessment under constant walking conditions, whereas sequence B was constructed for age estimation and gender classification.

### 4.4. OU-ISIR Multi-View Large Population Dataset

The OU-ISIR Multi-View Large Population dataset (OU-MVLP) comprised 10,307 individuals of which 5114 were males and 5193 were females. The individuals were from 2 to 87 years old. The gait sequences were recorded from 14 views in the range of 0–90° and 180–270°. The difference between each view was set to 15°. Two sequences were captured for each view angle for every individual. The dataset was intended for cross-view gait recognition, gender classification, and age estimation. A summary of gait datasets is presented in Table 5.

## 5. Conclusions

Gait recognition is one of the most rapidly evolving biometrics in recent years. Previously, handcrafted approaches had been applied to perform gait recognition. The handcrafted approaches can be categorized into model-based and model-free approaches. The model-based methods require more complicated computation, as they model the body parts and joints to track the body movement when walking. The model-free methods are also known as appearance-based methods, in which they directly capture the spatial and temporal features of the silhouettes in the gait cycle. The handcrafted approaches require manual feature engineering that is specifically tailored to the gait datasets. Due to this reason, the variation in walking style may greatly affects the extracted features, thus deteriorating the performance of handcrafted approaches. In view of this, the deep learning approaches were favoured by the researchers, which is attributable to the autonomous feature extraction and classification capability. The CNN models are the most popular methods in recent years as they are able to learn more complex features and capture the relationship between the features and classes. As gait is a continuous cycle over the time, RNN models are also leveraged to encode the long-term dependencies in the features. Among the commonly used gait datasets, the OU-MVLP dataset is relatively more challenging as it contains the largest number of subjects and the videos were captured from different angles. Motivated by the success of attention models, such as transformers, the future trends in gait recognition are likely to shift to attention models.

## Figures and Tables

**Table 1:** Summary of model-based approach.

Literature	Gait Features	Classifier	Dataset	Accuracy (%)
Ahmed et al. [30]	HDF and VDF	kNN	Own dataset	92
Wang et al. [31]	Static and dynamic parameters	NN	Own dataset	92.30
Sun et al. [32]	Static and dynamic features	NN	Own dataset	92.30
Zeng et al. [33]	Joint angles	Smallest error principle	CASIA-A	92.50
			CASIA-B	91.90
			CASIA-B	94
			CASIA-C	99
Deng et al. [34]	Lower limbs regions and lower limbs joint angles	Smallest error principle	TUM GAID	90
			OU-ISIR B	98
			USF HumanID	94.40
Sattrupai & Kusakunniran [35]	Motion trajectory, HOG, HOF and MBH (x,y)	kNN + Euclidean distance	CASIA-B	95
Kovic et al. [36]	Gait signals + LDA	kNN	OU-ISIR A	-
Sah & Panday [37]	CoB coordinates	Weighted-kNN	Own dataset	-
			CASIA-A	98.90
Sharif et al. [38]	Texture + shape + geometric	Euclidean distance	CASIA-B	95.80
			CASIA-C	97.30

**Table 2:** Summary of model-free approach.

Literature	Gait Features	Classifier	Dataset	Accuracy (%)
Jeevan et al. [44]	GPPE + PCA	SVM	CASIA-A	L-L: 73.68 L-R: 26.31
			CASIA-B	Nm: 93.36 Cl: 22.44 Bg: 56.12
			CASIA-C	Nm: 73.17 Bg: 43.74 Fast: 69.53 Slow: 56.95
			OU-ISIR A	100
Hosseini & Nordin [45]	Averaged silhouettes + PCA	Euclidean distance	TUM-IITKGP	60
Alvarez & Sahonero-Alvarez [46]	Modified GEI + PCA	LDA	CASIA-B	90.12
Luo et al. [48]	GEI + AFDEI	NN + Euclidean Distance	CASIA-B	Nm: 88.7 Cl: 91.9 Bg: 89.9
Arora & Srivastava [49]	GGI	NN + Euclidean Distance	CASIA-B	98
			Soton	100
Fathima et al. [50]	Kinematics parameters	SVM, kNN and RVM	CASIA-B	91.5
Rida et al. [51]	Scores of SD	1-NN + GLPP	CASIA-B	86.06
			CASIA-A	-
Wang et al. [52]	Gabor features + 2D2PCA	SVM	CASIA-B	93.52
			CASIA-C	-
Rida et al. [53]	Masked-GEI + PCA	MDA	CASIA-B	85.21
Rida [54]	Dynamic body parts	NN + CDA	CASIA-B	88.75
			CASIA-B	93.42
Mogan et al. [55]	MHI + BSIF + HOG	Euclidean distance + Majority voting	OU-ISIR D	DBhigh: 96 DBlow: 100
			CMU MoBo	76
			CASIA-B	97.37
Mogan et al. [56]	HTG	Euclidean distance + Majority voting	OU-ISIR D	DBhigh: 99 DBlow: 100
			CMU MoBo	92

**Table 3:** Summary of recurrent neural network approach.

Literature	Method	Dataset	Accuracy (%)
McLaughlin et al. [57]	CNN + RNN + Temporal pooling	iLIDS-VID	-
		PRID-2011	-
Varior et al. [58]	LOMO + CN	Market-1501	61.6
		CUHK03	57.3
		VIPeR	42.4
		Motion Capture Data AMC302.0	92.60
Li et al. [59]	Skeleton data	KINECTUNITO	97.33
		Kinect Gait Biometry	-
		CASIA-B	96.0
Zhang et al. [60]	Local + frame-level + weighted features	OU-ISIR LP	99.3
		OUMVLP	88.3
Battistone & Petrosino [61]	Changes of shape and size in graph	CASIA-B	87.8
		TUM-GAID	98.4
Tong et al. [62]	Spatial and temporal features	CASIA-B	-
Wang & Yan [63]	ff-GEI + CNN + LSTM	CASIA-B	95.9
		OU-ISIR LP	99.1
		Kinect Gait Biometry	97.39
Liu et al. [64]	SkeGEI features + DA features	SDU Gait	88.11
		CIL Gait	80.20
Zhang et al. [16]	Pose features + canonical features + appearance features	CASIA-B	Nm: 92.3 Bg: 88.9 Cl: 62.3
		USF	99.7
		FVG	91.3
Hasan & Mustafa [65]	2D body joints + joints angular trajectories + temporal displacement + body-part length	CASIA-A	-
		CASIA-B	Nm: 99.41 Bg: 97.80 Cl: 93.34
Li et al. [67]	HMR + CNN / LSTM	CASIA-B	Nm: 97.9 Bg: 93.1 Cl: 77.6
		OU-MVLP	95.8
Wen & Wang [68]	ff-GEIs + CNN + RLSTM	CASIA-B	-
		OU-ISIR LP	-

**Table 4:** Summary of convolutional neural network approach.

Literature	Method	Dataset	Accuracy (%)
Song et al. [69]	GaitNet	CASIA-B	92.6
Zhu et al. [71]	LFN (pre-processing included)	OU-LP	98.04
Su et al. [72]	CNN + Center-ranked loss	CASIA-B	Nm: 74.8
		OU-MVLP	57.8
Wen [73]	Gabor filter + CNN	CASIA-B	-
		OU-LP	-
Fan et al. [74]	FPFE + HP + MCM	CASIA-B	Nm: 96.2 Bg: 91.5 Cl: 78.7
		OU-MVLP	88.7
Hou et al. [75]	GLN	CASIA-B	Nm: 96.88 Bg: 94.04 Cl: 77.50
		OU-MVLP	89.18
Ding et al. [77]	SCN	CASIA-B	Nm: 95.2 Bg: 89.8 Cl: 73.9
		OU-MVLP	83.8
Yoo & Park [80]	Skeleton-based disentangled network	CASIA-B	Nm: 85.4 Bg: 77.4 Cl: 71.1
Jia et al. [81]	CNN + attention mechanism	CASIA-B	Nm: 92.48 Bg: 86.2 Cl: 68.74
Shiraga et al. [82]	GEINet	OU-LP	-
Yeoh et al. [83]	CNN	OU-ISIR Treadmill B	91.38
Alotaibi & Mahmood [87]	Deep CNN	CASIA-B	-
Wu et al. [88]	FBW-CNN	CASIA-B	37.9
		OU-LP	-
Khan et al. [89]	JIMEN + DN	OU-LP Bag	88.1
		OUTD-B	89.6
		TUM-GAID	63.5
Wu et al. [84]	LB & MT	CASIA-B	LB: 88.4 MT: 91.2
		OU-LP	94.8
Wang & Yan [92]	NLNN	CASIA-B	-
		OU-LP	-
Balamurugan et al. [93]	Deep CNN	CASIA-B	-
Wu et al. [90]	FWCN	CASIA-B	Nm: 88.62 Bg: 73.8 CL: 61.1
		OU-LP	-
Xu [91]	CNN (PST + RN)	CASIA-B	92.7
		OU-LP	98.93
		OU-MVLP	63.1
Elharrouss et al. [94]	Angle estimation CNN + Gait recognition CNN	CASIA-B	96.3
		OU-LP	-
		OU-MVLP	-
Takemura et al. [85]	3in (3 CNNs) + 2 diff (2 CNNs)	OU-LP	98.8
		OU-MVLP	52.7
Tong et al. [86]	Triplet-based CNN	CASIA-B	-
Xu [95]	DLMNN	CASIA-B	80.67
		OU-LP	-
Mogan et al. [96]	DenseNet-201 + MLP	CASIA-B	100
		OU-ISIR D	DBlow: 100
			DBhigh: 100
		OU-LP	99.17
Wang et al. [97]	Multichannel CNN	CASIA-A	-
		CASIA-B	-
		OU-LP	-
Wang & Zhang [98]	TCNN + SVM	CASIA-B	-
		OU-LP	-
Chao et al. [76]	GaitSet	CASIA-B	Nm: 96.1 Bg: 90.8 Cl: 70.3
		OU-MVLP	87.9
Liu & Liu [99]	TS-Net	CASIA-B	Nm: 68.4 Bg: 58.4 Cl: 41.9
		UCMP-GAIT	92.22
Chai et al. [100]	Backbone + HPP + HPM	CASIA-B	Nm: 95.6 Bg: 89.2 Cl: 73.4
		OU-MVLP	89.9
Wang & Yan [101]	GCF-CNN	CASIA-A	65.64
		CASIA-B	62.36
		OU-LP	64.33

**Table 5:** Summary of Gait Datasets.

Datasets	Number of Subjects	Variations
CASIA-B	124	Normal walking Clothing Carrying condition
OU-ISIR D	185	Steady walking Fluctuated walking
OU-LP	4016	4 viewing angles
OU-MVLP	10,307	14 viewing angles

## Data Availability

Not applicable.
